# “Begging the Question”—Does *Toxocara* Infection/Exposure Associate with Multiple Sclerosis-Risk?

**DOI:** 10.3390/pathogens9110938

**Published:** 2020-11-11

**Authors:** Ali Taghipour, Ali Rostami, Sahar Esfandyari, Saeed Aghapour, Alessandra Nicoletti, Robin B. Gasser

**Affiliations:** 1 Department of Parasitology, Faculty of Medical Sciences, Tarbiat Modares University, Tehran, Iran; alitaghipor71@yahoo.com; 2 Infectious Diseases and Tropical Medicine Research Center, Health Research Institute, Babol University of Medical Sciences, Babol, Iran; 3 Immunoregulation Research Center, Health Research Institute, Babol University of Medical Sciences, Babol, Iran; 4 Department of Anatomy, School of Medicine, Tehran University of Medical Sciences, Tehran, Iran; saharesfandyari1366@gmail.com; 5 Department of Neurosurgery, Faculty of Medicine, Mazandaran University of Medical Sciences, Sari, Iran; aghapour.saeed@yahoo.com; 6 Department G.F. Ingrassia, Section of Neurosciences, University of Catania, 95123 Catania, Italy; anicolet@unict.it; 7 Department of Veterinary Biosciences, Melbourne Veterinary School, The University of Melbourne, Parkville 3010, VIC, Australia

**Keywords:** *Toxocara*, multiple sclerosis, association, meta-analysis

## Abstract

Although the cause of multiple sclerosis (MS) is unclear, infectious agents, including some parasitic roundworms (nematodes), have been proposed as possible risk factors or contributors. Here, we conducted a systematic review and meta-analysis of published observational studies to evaluate whether there is a possible association between infection with, or exposure to, one or more members of the genus *Toxocara* (phylum Nematoda; superfamily Ascaridoidea) and MS. We undertook a search of public literature databases to identify relevant studies and then used a random-effects meta-analysis model to generate the pooled odds ratio (OR) and 95% confidence intervals (CIs). This search identified six of a total of 1371 articles that were relevant to the topic; these published studies involved totals of 473 MS patients and 647 control subjects. Anti-*Toxocara* IgG serum antibodies were detected in 62 MS patients and 37 controls, resulting in respective seroprevalences of 13.1% (95% CI: 8.2–20.3) and 4.8% (95% CI: 2.5–9.2), indicating an association (pooled OR, 3.01; 95% CI: 1.46–6.21). Because of the publication bias identified (six eligible studies), well-designed and -controlled studies are required in the future to rigorously test the hypothesis that *Toxocara* infection/exposure has an association with MS.

## 1. Introduction

Over the past decade, the World Health Organization (WHO) has emphasized the major importance of investigating neurological disorders in humans [1]. Key disorders, such as multiple sclerosis (MS) as well as Alzheimer’s and Parkinson’s diseases, cause major morbidity and mortality worldwide [1]. MS is a prevalent, chronic, and immune-mediated disease of the central nervous system (CNS), causing significant neurological disability worldwide [2,3]. According to the Global Burden of Disease Study [4], MS contributes to >1.151 million disability-adjusted life-years (DALYs) annually, and was linked to 18,932 deaths in 2016. While the exact cause(s) of MS is (are) not yet understood, the underlying mechanism is thought to be autoimmunity and/or a failure of particular cells to produce myelin [5,6]. Multiple factors, including genetic background, immune dysregulation, and environment, are proposed to contribute to the aetio-pathogenesis of this disease [7,8,9].

Studies have investigated the possible roles of infectious agents, mainly viruses, of which Epstein-Barr virus (anti-EBNA IgG sero-positivity) and infectious mononucleosis were reported to have a positive association with MS [10]. On the other hand, according to the ‘hygiene hypothesis’, multiple infectious exposures in early childhood reduce the risk of autoimmune and allergic diseases, as is commonly observed in tropical and subtropical areas [11]. The hygiene hypothesis has been proposed to explain an apparent increase in MS in Western countries, with an imbalance between Th1 and Th2 immune responses being the immunological reasoning [12]. In accord with this hypothesis, the modulation of autoimmune responses by some helminths has been shown to associate with several autoimmune diseases [13,14,15], and it has been proposed by some workers that helminths could have a protective effect against MS via a down-modulation of inflammatory responses and an enhancement of immune regulation [16]. Although some experimental evidence supports a protective effect, the relationship between helminths and MS is still a matter of major contention [17]. Indeed, some helminths (e.g., *Necator americanus* and *Trichuris suis*) appear to reduce the risk of MS [18,19], while others, such as *Toxocara* spp., may contribute to MS development [20,21].

*Toxocara* infection of humans is caused by the larval stages of members of the genus *Toxocara*, principally *T. canis*, but sometimes *T. cati* or related species from canids (dogs) or felids (cats) [22,23,24,25]. It is estimated that >1.4 billion people worldwide are infected with, or exposed to, *Toxocara* spp., indicating that infections are widespread, and thus, likely being responsible for human toxocariasis [25,26]. Human infection occurs via the ingestion of *Toxocara* eggs (containing infective third-stage larvae) from contaminated soil or raw vegetables, or sometimes via eating undercooked or raw meat (e.g., chicken and lamb) from paratenic hosts carrying encysted *Toxocara* larvae [27,28]. Following the ingestion of eggs or larvae, individual larvae emerge/activate in the small intestine, penetrate the gut wall and then migrate to different organs via the systemic circulation, but do not develop to mature adult worms in the gut [29]. The migrating larvae can cause significant damage to multiple organ systems in the accidental human host, such as the viscera and nervous system (including eyes), which can lead to disease and permanent damage [28,30].

*Toxocara* larvae can cross the blood-brain barrier to invade the CNS and cause neurotoxocariasis [31], although the detection of such larvae in brain tissues can be challenging [32,33]. Larval migration induces a host response, characterized mainly by a T-helper cell (Th2) response, cellular infiltration around larvae, and increased production of cytokines (interleukins-4, -5, -10 and -13), peripheral eosinophilia and/or specific serum IgG and IgE antibodies [29,34]. The neurological manifestation of neurotoxocariasis is variable and can include encephalitis, meningitis, myelitis, and/or cerebral vasculitis, but asymptomatic CNS infection is common [31,35,36]; MRI findings of neurotoxocariasis include subcortical, cortical or deep white matter lesions with variable enhancement, which can associate with hydrocephalus and leptomeningeal enhancement, as well as spinal cord involvement [37]. Human neurotoxocariasis is thought to be rare, even if, in many animal models (e.g., rodents, pigs, and primates), *Toxocara* larvae usually migrate to the brain [38]. Although toxocariasis is a prevalent helminthiasis worldwide, characterized by a pronounced Th2 host response [29], few studies have evaluated its possible role on the risk of MS [20,21,39,40], and no study has yet been carried out to systematically review existing data and information on this topic. This study scrutinizes all publicly available peer-reviewed literature to critically evaluate whether human *Toxocara* infection/exposure associates with the risk of MS, or not.

## 2. Materials and Methods

The Preferred Reporting Items for Systematic Reviews and Meta-analyses (PRISMA) guidelines [41] were followed for the present study design, as well as for the analysis and interpretation of results.

### 2.1. Search Strategy and Study Selection

A comprehensive search of the literature was performed using five international databases, including PubMed, Scopus, Science Direct, Web of Science, and Google Scholar, from inception to 15 June 2020. The search terms followed medical subject headings (MeSH): “*Toxocara* infection”, “toxocariasis”, “*Toxocara canis*”, “*Toxocara cati*”, “multiple sclerosis”, “association”, and “relationship”, alone or in combination with “OR” and/or “AND”. The reference lists in the eligible studies were used to access relevant, related studies and to optimize data acquisition. After completing the search, the articles selected were independently reviewed by the two researchers (A.T. and A.R.). Following an appraisal of the title, abstract, and full text of individual articles, all duplicates or publications unrelated to the topic were excluded. The full texts of relevant publications were read and scrutinized to identify articles eligible for inclusion, and any conflicts of opinion or uncertainties were resolved through detailed discussions, and a consensus was reached. Included were peer-reviewed, original or conference papers describing: (1) Cross-sectional, cohort or case–control studies; (2) prevalence studies of humans for *Toxocara* infection or exposure, with both MS and suitable control groups; (3) serological or histopathological investigations of human *Toxocara* infection or toxocariasis; (4) published (up to 15 June 2020), without applying a language, time or geographic limitation. Excluded were articles: (1) Without one or more appropriate control groups of healthy people; (2) with a sample size of ≤30 in each group; (3) experimental studies; and (4) reviews, case reports, and letters without original research results or data sets.

### 2.2. Data Extraction and Quality Assessment

Eligible articles were individually scrutinized, and data/information extracted. The data recorded were: First author, publication year, country, diagnostic methodology, age (mean or range) and gender of human subjects, total numbers of MS patients and healthy control subjects, as well as the prevalence of anti-*Toxocara* serum antibodies in individuals of each of the subject groups. The quality of each eligible article was assessed using the Newcastle–Ottawa Scale (NOS), as recommended by the Cochrane network [42,43]. The scoring system was: Subject selection criteria (0–4 points), comparability of subjects (0–2 points), and exposure (0–3 points)—with a nine-point maximum. An article was given one star for each numbered item meeting the selection and exposure criteria, and two stars were given for comparability. Using the sum of all points, the quality of each article was rated as high (7–9 points), moderate (4–6), or poor (0–3).

### 2.3. Data Synthesis and Statistical Analysis

All statistical analyses were conducted using comprehensive meta-analysis software (version 2, BIOSTAT, Englewood, NJ, USA). First, we estimated the pooled prevalence of *Toxocara* infection/exposure with a corresponding 95% confidence interval (CI) in each case and control groups employing the DerSimonian-Laird random-effects model, and the difference was calculated by χ^2^ test. We used the exact binomial method of Hamza et al. to model within-study variability by binomial distribution and Freeman-Tukey Double Arcsine Transformation to stabilize the variances in the meta-analysis [44]. Then, the respective odds ratio (OR) and 95% CI was calculated for each article/study. To assess an association between *Toxocara* exposure/infection and MS in humans, ORs from individual studies were combined to produce a pooled OR and 95% CI, employing the random-effects model with a restricted maximum-likelihood estimator. Heterogeneity among studies was assessed using *I*^2^ and Cochran’s Q statistics [45]. The effects of a small study and of publication bias on results were inferred using the Egger’s regression test [46]. A *p*-value of < 0.05 was considered statistically significant.

## 3. Results

**Figure 1:**
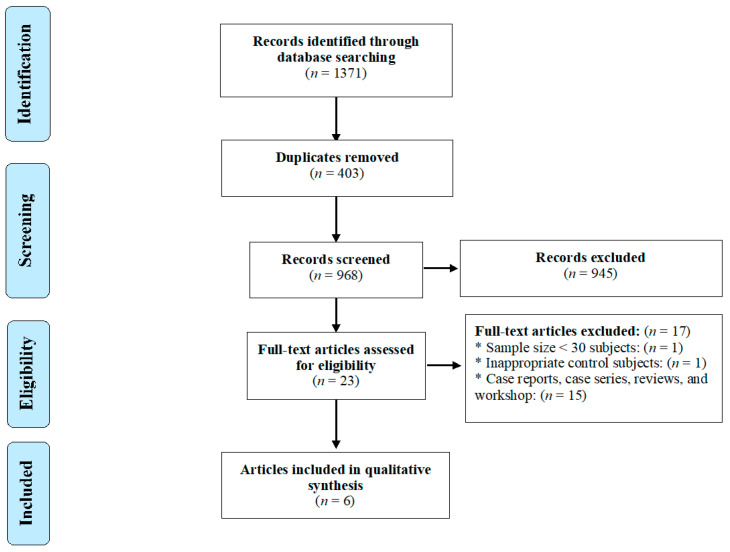
PRISMA flow diagram of the search strategy and study selection process.

The systematic search identified 1371 articles of possible relevance, 8 of which remained and were pertinent, following duplicate-removal, title- and abstract-screening, and implementation of all eight inclusion and exclusion criteria (Figure 1). Of the eight articles selected, two [21,47] were excluded. The first article [21] was removed because of its small sample size (<30 in both the case- and control-groups) and sero-negative results for all participants in both groups; the second [47] was eliminated, due to the recruitment of inappropriate control subjects (i.e., patients with clinically isolated syndrome [CIS]—a first neurological episode of MS). In this latter study, serum IgG-antibodies were detected in ELISA using against *Toxocara* excretory/secretory (TES) antigens in one of 62 (1.6%) MS patients, whereas none of the CIS patients had measurable titers.

Therefore, six studies [20,39,40,48,49,50] qualified and were, ultimately, included in the meta-analysis (Figure 1). Five studies were original papers, published in peer-reviewed journals, and one [49] was a conference paper. These studies had a case–control design, were from Iran (*n* = 4), Italy (*n* = 1) and Turkey (*n* = 1), and were published between 2006 and 2020 (Table 1). The key characteristics of each of these studies are listed in Table 1, and the results of the quality assessment of case–control studies are given in Table 2.

The six articles included in the present meta-analysis showed an acceptable quality (i.e., had a high or moderate quality-score; cf. Table 2). All of them reported prevalences of anti-*Toxocara* IgG serum antibodies, established by enzyme-linked immunosorbent assay (ELISA), although results were divergent between studies; three reported a non-significant, while the three others reported a significant, positive association between *Toxocara* infection/exposure and MS (Table 1).

**Figure 2:**
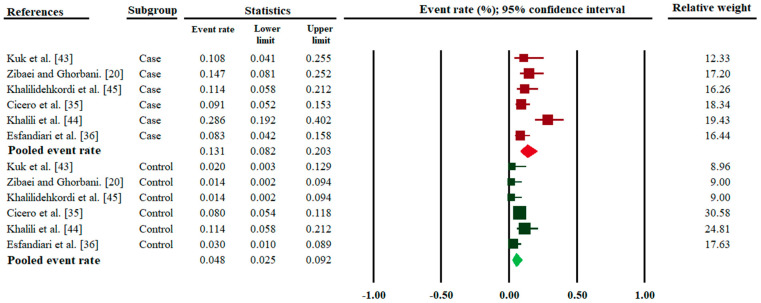
Forest plots for random-effects meta-analysis of the prevalence rates of *Toxocara* infection/exposure (established by anti-*Toxocara* IgG serum antibody detection) in MS patients (cases) and in healthy control subjects (controls). Relative weight: Weight of each study by comparison with all six studies—in percent.

In total, 473 MS patients were recruited to all six studies selected; 62 subjects had anti-*Toxocara* IgG serum antibodies. Of the 647 control subjects, 37 people had anti-*Toxocara* IgG serum antibodies. The pooled anti-*Toxocara* sero-prevalence rate in MS patients (13.1%; 95% CI, 8.2–20.3; *I*^2^ = 71.1; 95% CI, 32.7 to 87.5; Q-value = 17.325) was higher (*p* value < 0.01) than in the control group (4.8%; 95% CI, 2.5–9.2; *I*^2^ = 57.4; −5.3 to 82.6; Q-value = 11.83) (Figure 2).

**Figure 3:**
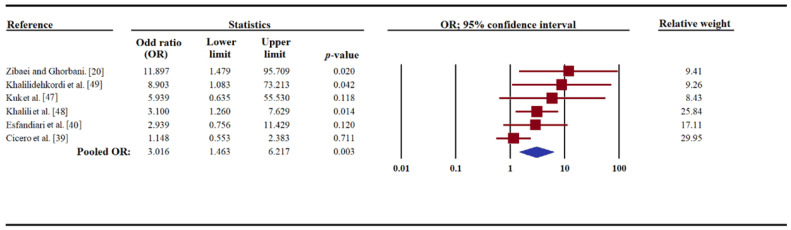
Forest plot, pooled with random-effects regarding the association between *Toxocara* infection/exposure (assessed by anti-*Toxocara* IgG serum antibody detection) and multiple sclerosis (MS), showing the odd ratio (OR) and a 95% confidence interval (CI). The *p*-value referred to the significance of OR.

The results of the meta-analysis of all six studies showed a pooled OR of 3.01 (95% CI, 1.46–6.21), suggesting that *Toxocara* infection/exposure could be significantly associated with an increased risk of MS (Figure 3). Some heterogeneity (*I*^2^ = 42.4; 95% CI, −45.2 to 76.1; Q-value = 8.68) was detected among studies. Sensitivity analysis was performed to determine the effect of one study (reported in a conference paper) on our estimated OR; this analysis showed that, after removing this study [49], the association was still significant (OR, 2.6; 95% CI, 1.2–5.6; *I*^2^ = 43.2; 95% CI, −53.8 to 78.40; Q-value = 7.05) (Figure S1). In one of the six studies included [49], based on the proportion of anti-*Toxocara* sero-positive patients, women were significantly associated with developing MS. For two [20,40] of the six studies, there was no significant difference in gender between the MS patient- and healthy control groups. Employing the Egger’s regression test, a significant publication bias was found in the studies included here (*t*-value = 3.53, *p*-value = 0.02; Figure S2).

## 4. Discussion

The hygiene hypothesis has been proposed as a possible explanation for the increased incidence of allergy and autoimmune diseases, including MS, in the Western world, with an imbalance between Th1 and Th2 responses being promoted as an immunological explanation [12]. Epidemiological studies have indicated that the prevalence of MS has been increasing over time, particularly in countries whose socioeconomic and sanitation levels have improved, probably through a progressive decrease in the prevalence of infections [51]. Some helminth species have been reported or proposed to have a role in preventing autoimmune diseases. Supporting this hypothesis are some experimental studies showing that parasites, such as *Schistosoma mansoni*, exert a protective effect on the development of experimental allergic encephalomyelitis (EAE) in infected mice, dampening the classical Th1 response through an immunological switch to a Th2 response [52]. However, clearly, the possible relationship between helminths and MS is still controversial. On the one hand, for instance, *Necator americanus* larvae [18] and *Trichuris suis* eggs [19] are proposed to play a role in protecting people against MS. On the other hand, some other worms, such as *Toxocara* species [20] and *Onchocerca volvulus* [53], have been suggested to contribute to autoimmune diseases [21].

The present meta-analysis provides epidemiological evidence of a significant association between *Toxocara* infection/exposure and MS, with a pooled overall OR of 3.01 (95% CI, 1.46–6.21). The results of this study accord with those reported in some systematic reviews and meta-analyses examining the role of *Toxocara* infection as a potential risk factor for other neurological syndromes, such as epilepsy [54,55].

*Toxocara* larvae can migrate in the tissues of the central nervous system (CNS) and induce the neural larva migrans (NLM) syndrome [35,56]. At the end of the visceral phase of migration, *Toxocara* larvae commence the myotropic-neurotropic phase of migration, and reach the brain within 28 days of infection [56]. An experimental study of NLM in mice [57] showed that larvae penetrate arteries, near the brain surface, and assume a predilection for the cerebellum, rather than the cerebrum or brainstem. *Toxocara* larvae might survive for up to two years after infection in the brains of experimentally infected mice [58]. Furthermore, CNS invasion of *Toxocara* larvae results in parenchymal damage, hemorrhagic lesions, demyelination, focal malacia, and neuronal necrosis in the brain [59,60,61,62,63].

Of the animal/toxocariasis models employed, the pig model has been particularly useful because of physiological and biochemical similarities between pigs and humans, especially in relation to immune responses. In pigs, *Toxocara* larvae can be recovered from the brain between days 10–21 after infection, but disease in the pig is self-limiting, and larvae become undetectable in the brain after a period of 120 days. Pathological changes associated with porcine cerebral toxocariasis include congestion, oedema, shrinkage of nerve cells, vacuolization, gliosis, satellitosis, neurophagia, and liquefactive necrosis [38]. However, it should be noted that, according to a recent study in pigs [64], the passage of *Toxoxara* larvae through the brain does not always induce lesions detectable by magnetic resonance imaging (MRI), suggesting that they do not cause structural lesions, thus leaving no detectable damage.

Despite this evidence, there is a ‘missing link’ between *Toxocara* infection/exposure and the pathogenesis of MS in humans, which warrants serious attention [65]. Some pathogenic and immunologic mechanisms have been suggested to explain the possible implication(s) of toxocariasis or *Toxocara* infection in MS: (1) While demyelination and neurodegeneration processes are key characteristics of MS [5], they are also frequently-observed histopathological hallmarks of NLM and neurotoxocariasis in mice with experimental *T. canis* infection [60,61,63,66,67]. Here, demyelination might relate to a reduced cholesterol concentration or a down-regulation of key genes involved in cholesterol synthesis or transport via alterations in signal transduction induced by the presence of *Toxocara* [56,63]. (2) Experimental studies of mice have shown that migrating *Toxocara* larvae can be responsible for increased permeability of the blood-brain barrier, and an elevated expression of nitric oxide synthase (iNOS) and pro-inflammatory cytokines, such as interleukin−1β, interleukin −6 and tumor necrosis factor-α, which are potentially neurotoxic substances, and could lead to neurodegeneration or neuronal damage [35,68]. Although these pathological and immunological observations attempt to explain a possible association of *Toxocara* infection/exposure and/or toxocariasis with MS, it is important to point out: (i) That many observations have been made in mice experimentally infected with *T. canis* by comparison with matching, uninfected control mice, and (ii) that, although both humans and mice are accidental hosts, the immunological responses in experimentally infected mice could be distinct from those in people who contract accidental *Toxocara* infection. Other factors to consider (in relation to both host species) would be the intensity of the larval infection/burden and different immune responses to these larvae among individuals (i.e., high responders *versus* low responders). As mouse models exist for human toxocariasis [30,56,69], it would be informative to conduct systematic, comparative studies to critically assess experimentally whether *Toxocara* infection/exposure contributes to MS, but it will be important to evaluate which experimental model should be used to answer which specific question, and ensure cautious interpretation of experimental findings for mice with respect to MS in humans.

This study is the first systematic review and meta-analysis of all publicly accessible, published data/information to assess an association between human *Toxocara* infection/exposure, and MS risk. Although the present literature search was comprehensive and the methodology rigorous, the results of this meta-analysis need to be interpreted with caution, mainly for the following reasons: (1) All of the studies included here are retrospective case–control studies, such that one cannot be confident that *Toxocara* exposure or infection occurred before the outcome (i.e., MS), and consequently, a possible ‘reverse causality’ cannot be excluded; (2) most studies selected were hospital-based, such that selection bias could not be ruled out; (3) the numbers of studies (*n* = 6) and participants were limited, and from a small number of countries; (4) some studies published in local journals (not indexed in global databases) might have been missed in the literature search; (5) human case- and control-groups were not adjusted for different factors, such as age or gender; (6) potential risk factors (pica or exposure to pets) were not systematically evaluated; (7) in most articles, the definition of a ‘control group’ was unclear; (8) there was a publication bias, such that that studies with a significant difference between MS patients and controls were probably more likely to be published; and (9) eligible studies included in the meta-analysis had used only ELISA to detect anti-*Toxocara* antibodies, and did not employ a confirmatory method (e.g., immunoblotting) to exclude cross-reactivity with serum antibodies against other nematodes, such as *Ascaris*.

## 5. Conclusions

Although the small number of published studies (*n* = 6) investigating whether *Toxocara* infection/exposure could be associated with MS limit the interpretations of the findings and the conclusions that could be drawn from the meta-analysis, the present study emphasizes the need for well-designed and well-controlled longitudinal (cohort) studies in the future, to rigorously test the hypothesis that *Toxocara* infection/exposure has an association with MS, and to assess whether such infection/exposure is a co-factor contributing to the development of MS.

## Figures and Tables

**Table 1:** Key characteristics of studies used to investigate an association between human *Toxocara* infection/exposure and multiple sclerosis (MS).

Reference	Country	Age Range, or Mean Age ± Standard Deviation (Years)		Subjects with MS		Subjects without MS (Controls)		*p*-Value
		Subjects with MS	Subjects without MS Controls	Number Tested	Positive for Anti-*Toxocara* IgG Serum Antibodies (%)	Number Tested	Positive for Anti-*Toxocara* IgG Serum Antibodies (%)	
Kuk et al. [47]	Turkey	20–54	20–50	37	4 (10.8)	50	1 (2.0)	0.08
Zibaei and Ghorbani. [20]	Iran	3–49	3–52	68	10 (14.7)	70	1 (1.4)	0.004
Khalilidehkordi et al. [49]	Iran	not recorded	not recorded	70	8 (11.4)	70	1 (1.4)	<0.05
Cicero et al. [39]	Italy	44.6 ± 11.1	48.1 ± 15.6	132	12 (9.1)	287	23 (8.0)	0.7
Khalili et al. [48]	Iran	41.2 ± 9.5	38.8 ± 7.6	70	20 (28.5)	70	8 (11.4)	0.02
Esfandiari et al. [40]	Iran	11–60	11–60	96	8 (8.3)	100	3 (3.0)	0.1

**Table 2:** Newcastle–Ottawa Scale for assessing the quality of the six case–control studies included to assess an association between human *Toxocara* infection/exposure and multiple sclerosis (MS).

	Selection				Comparability	Exposure			
Reference	Adequate Case Definition	Representat-Iveness of MS Cases	Selection of Controls	Definition of Controls	Comparability of Cases and Controls on the Basis of Design or Analysis	Ascertainment of Exposure	Same Method of Ascertainment for Cases and Controls	Non-Response Rate	Score
Kuk et al. [47]	*	*	*	na	*	*	*	na	6
Zibaei and Ghorbani. [20]	*	*	*	*	**	*	*	na	8
Khalilidehkordi et al. [49]	*	*	*	na	na	*	*	na	5
Cicero et al. [39]	*	*	*	*	**	*	*	*	9
Khalili et al. [48]	*	*	*	na	**	*	*	na	7
Esfandiari et al. [40]	*	*	*	*	**	*	*	na	8

In this table, one star was given to each article for each item meeting the selection and exposure criteria, and two stars were given for comparability. Using the sum of all points, the quality of each article was rated as high (7–9 points), moderate (4–6), or poor (0–3); not applicable (na).

## Supplementary Materials

The following are available online at https://www.mdpi.com/2076-0817/9/11/938/s1. Figure S1: Forest plot for sensitivity analysis (after removing of study by Khalilidehkordi et al. [49], which was a conference paper) pooled with random-effects regarding the association between *Toxocara* infection/exposure (assessed by anti-*Toxocara* IgG serum antibody detection) and multiple sclerosis (MS), showing the odd ratio (OR) and a 95% confidence interval (CI). Figure S2: Publication bias, calculated using an Egger’s plot.
