# Supplementary material for: “Begging the Question”—Does Toxocara Infection/Exposure Associate with Multiple Sclerosis-Risk?
Source: Pathogens. 2020 Nov 11;9(11):938. doi: 10.3390/pathogens9110938 (PMC7696196; doi:10.3390/pathogens9110938)
Supplement: Supplementary file 1 [file pathogens-09-00938-s001.pdf]

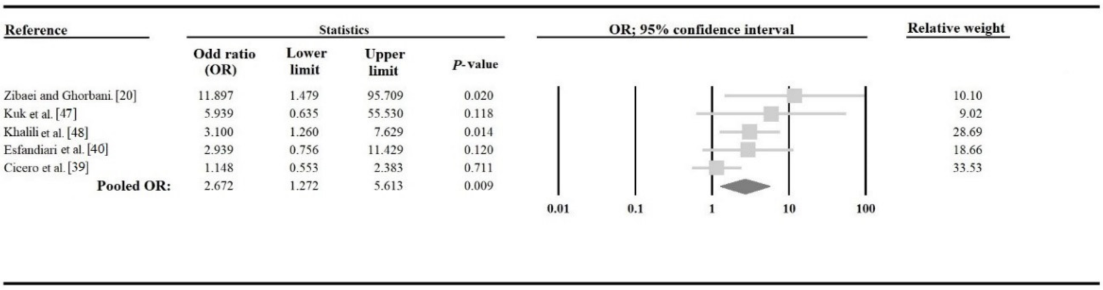

Figure S1. Forest plot for sensitivity analysis (after removing of study by Khalilidehkordi et al. [49], which was a conference paper) pooled with random effects regarding the association between *Toxocara* infection/exposure (assessed by anti-Toxocara serum IgG antibody detection) and multiple sclerosis (MS), showing the odd ratio (OR) and a 95% confidence interval (CI). The *P*-value referred to the significance of OR.

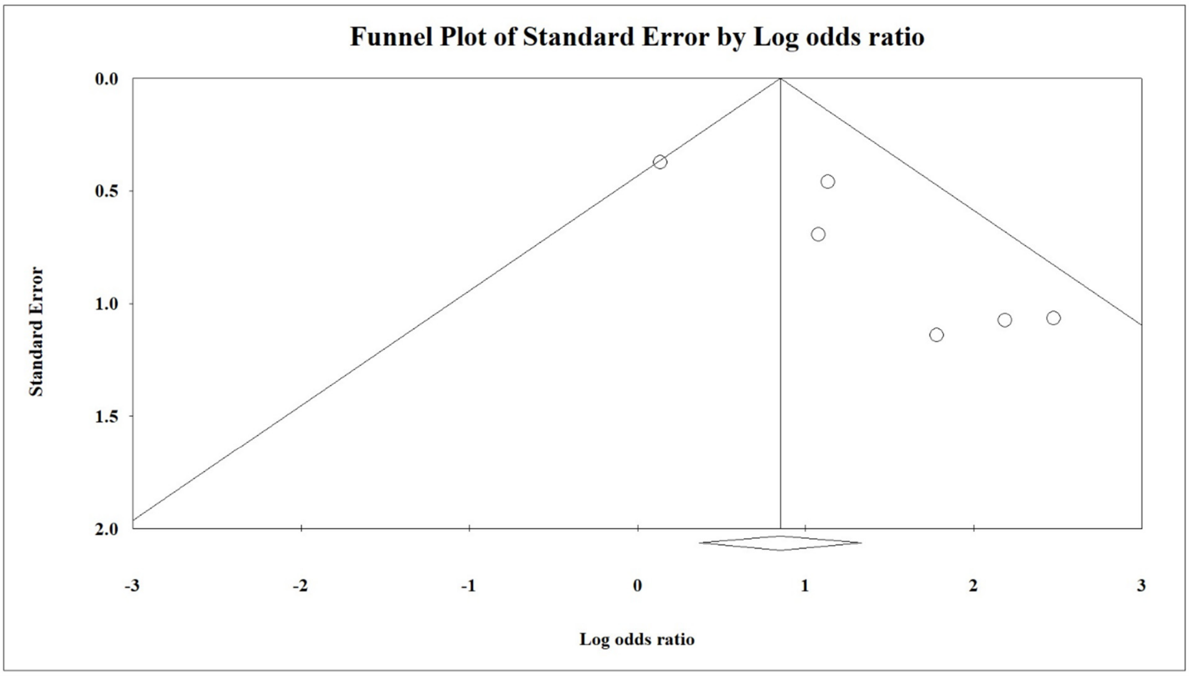

Figure S2. Publication bias calculated using an Egger's plot.
